# Clinical development of photodynamic agents and therapeutic applications

**DOI:** 10.1186/s40824-018-0140-z

**Published:** 2018-09-26

**Authors:** Rengarajan Baskaran, Junghan Lee, Su-Geun Yang

**Affiliations:** World Class Smart Lab, Department of New Drug Development, Inha University College of Medicine, 366, Seohae-daero, Jung-gu, Incheon, 22332 Republic of Korea

**Keywords:** Photodynamic therapy, Photosensitizers, Porphyrins, Clinical trials, Clinical unmet need

## Abstract

**Background:**

Photodynamic therapy (PDT) is photo-treatment of malignant or benign diseases using photosensitizing agents, light, and oxygen which generates cytotoxic reactive oxygens and induces tumour regressions. Several photodynamic treatments have been extensively studied and the photosensitizers (PS) are key to their biological efficacy, while laser and oxygen allow to appropriate and flexible delivery for treatment of diseases.

**Introduction:**

In presence of oxygen and the specific light triggering, PS is activated from its ground state into an excited singlet state, generates reactive oxygen species (ROS) and induces apoptosis of cancer tissues. Those PS can be divided by its specific efficiency of ROS generation, absorption wavelength and chemical structure.

**Main body:**

Up to dates, several PS were approved for clinical applications or under clinical trials. Photofrin® is the first clinically approved photosensitizer for the treatment of cancer. The second generation of PS, Porfimer sodium (Photofrin®), Temoporfin (Foscan®), Motexafin lutetium, Palladium bacteriopheophorbide, Purlytin®, Verteporfin (Visudyne®), Talaporfin (Laserphyrin®) are clinically approved or under-clinical trials. Now, third generation of PS, which can dramatically improve cancer-targeting efficiency by chemical modification, nano-delivery system or antibody conjugation, are extensively studied for clinical development.

**Conclusion:**

Here, we discuss up-to-date information on FDA-approved photodynamic agents, the clinical benefits of these agents. However, PDT is still dearth for the treatment of diseases in specifically deep tissue cancer. Next generation PS will be addressed in the future for PDT. We also provide clinical unmet need for the design of new photosensitizers.

## Background

Photodynamic therapy is a minimal-invasive combinatorial therapeutic modality with advantages of normal tissue preservation, relatively less pain and clinically approved for early stage disease in particularly for cancer [1–4]. Photodynamic therapy (PDT) has proven to exert specific cytotoxicity to tumour leading to cell death. The application of PDT is not only for oncology, but also being explored with different type of disease such as dermatology, cardiovascular and ophthalmology [5, 6]. PDT can be performed in the presence of oxygen, specific wavelength of light and photosensitizer (PS). Upon the light absorption, the PS transforms from ground state to an excited single state. This excited state produces radical and reactive oxygen species (ROS) [7, 8]. The ROS (i.e OH, O_2_ and H_2_O_2_) that cause the cellular damage leads to kill tumour through necrosis or apoptosis. PDT agent are pharmacologically inactive until they are exposed to light in the presence of oxygen.

Light therapy has been used for several thousand years, since the Ancient, Indian and Chinese civilization for the treatment of various diseases [9]. In 1897, chemical sensitizer was first published observation of photosensitizing effect in tissue by light source [10]. The first modern light therapy was reported in 1903 by Finsen who received the Nobel prize and used topically applied eosin and white light to treat skin cancer [2, 11]. The first PDT was tried for bladder cancer in 1976 [12], and other study was conducted for skin and lung tumours were efficient enough to control cancer growth [13, 14]. The photofrin, first PDT reagent, was approved in 1993 for the bladder cancer treatment. Currently, photofrin has been approved for various type of cancer by Food and Drug Administration (FDA).

Over the decades, number of studies related to the PDT have been performed for various types of cancer treatment, and few studies were attempted on the human immune system. PDT can be also recommended for pre-malignant type of cancers. However, PDT has disadvantages of photosensitive side effects; inconvenience, and relatively high cost, etc. Light penetrates up to less than centimeter length, and it is difficult to cover large areas. Heterogeneity of response from the variant light penetration depth is known to be another limitation of PDT. In this review, we explore the PDT techniques such as PS, light source and future direction for the treatment of cancer with clinically available PS and potential strategies for enhanced photodynamic effects.

## Principle and mechanism of PDT

**Fig. 1:**
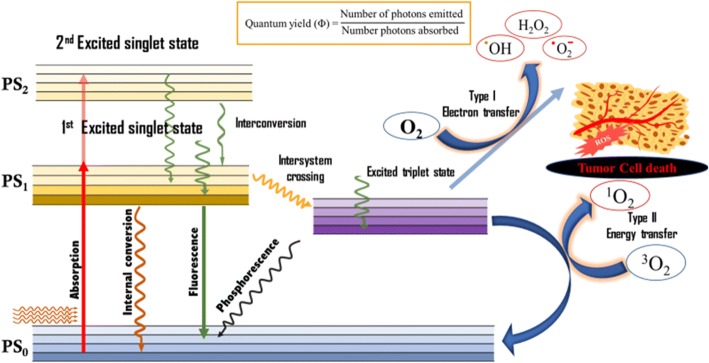
Modified Jablonski diagram depicting the process of photodynamic therapy

PDT works with three keys such as oxygen, light and PS. PDT possesses a multiple-stage process; administration of a PS, selective accumulation of PS to target tumour followed by illumination of light in target site. An appropriate wavelength of light should be selected for the full activation of the PS. Principle of photodynamic and electronic excitation of molecule is explained in modified jablonski diagram shown in Fig. 1 [15, 16]. The PS are transferred from its ground state into an excited singlet state under the specific wavelength of light. In presence of oxygen, the excited PS can react with substrates forms radicals or radical ions. Excited triplet state reaction occurs in two types (Fig. 1) (i.e. Type I and II) and generates active radical that causes the cellular damage and tissue necrosis or apoptosis. Type I pathway occurs when the excited molecules reacts with substrates and it produces cytotoxic species of radicals or radical ions. The excited triplet state PS reacts with molecular oxygen generating singlet oxygen by the energy transfer (Type II) [17].

Normal human tissues are exposed to oxygen levels about 5% (~ 40 mmHg) which is lower than inspired air. However, oxygen lever may differ based on blood haemoglobulin content. Some of researcher was reported that cancer cells may have low oxygen level and grow faster [18–21]. To overcome this issue nanoparticle or drug delivery system, which can produce singlet oxygen in the presence of light will be another tool for successful PDT. Co-administration of multiple target nanoparticle will be considered in future generation PS for the treatment of cancer. PS selectively up-taken by tumour tissue can escape cellular damage to the normal tissue. The benefit of PDT depends on the nature, property and localization of PSs and illumination conditions.

## Light source

The clinical efficacy of PDT is dependent on dose, exposure time, and delivery mode of light. However, penetration depth is regarded as crucial factor for therapeutic efficacy of PDT. There are several light sources used for PDT, such as ultraviolet light (330-400 nm), red light (600–700 nm) and near infrared (NIR) light (700–1000) [22]. There were different light sources applied based on the PS. However, the PS research are not precise for the treatment of cancer and other disease. If, PS itself works effectively, it would be milestone in cancer research. There was none of literature specified to kills the cancer cells with specific wavelength of light source. Most of the PDT studies were reported that PS was administered intravenous or oral injection and after several hours or days, the tumour is exposed to non-thermal light of the specific wavelength and PS will be activated and kills cancer cells. Light penetration was reported to be ~ 3 mm underneath of the skin in clinical study. Red and infrared light are penetrating deep tissue than short wavelength. Consequently, longer wavelength light penetrates deep into the tissue and damage the tumour cells [23]. Photo-bleaching can be another issue of PDT treatment. And single shot light source even in the similar clinical case of condition and indication is not always ideal for the PDT, even the same source of PS was applied.

## PSs for anticancer PDT

**Fig. 2:**
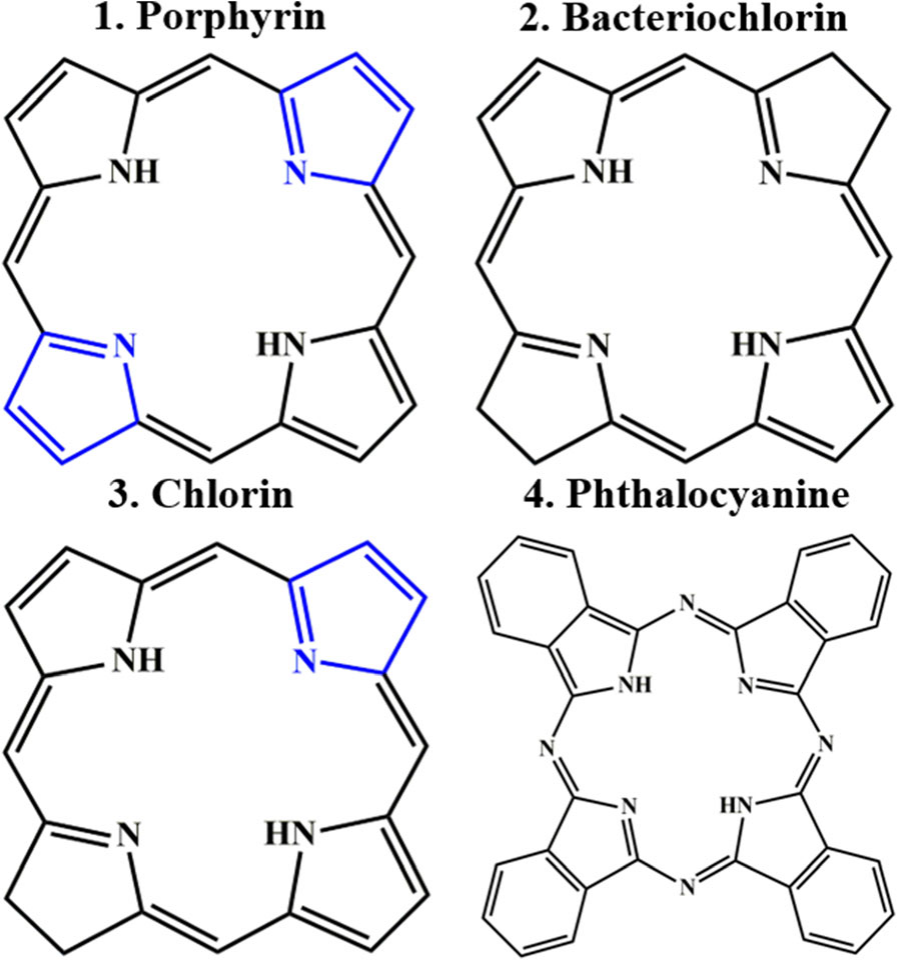
Basic structure of PSs. 1. Porphyrin (ex: 407 nm, em 620 nm); 2. Bacteriochlorin (ex: 374 nm, em 795 nm); 3. Chlorin (ex: 405 nm, em 670 nm); 4. Phthalocyanine (ex: 635 nm, em 700 nm)

PSs which generates triplet excited state of energy in the presence of light source are another key factor in photodynamic therapy. After the PDT patients should be warned to avoid exposure of skin and eyes to direct sunlight. Some of PS might last for more than 3 months and patients instructed to avoid day light and wear protective clothing and dark sunglasses when outdoors. Patients should be encouraged to stay at ambient indoor light to facilitate elimination of PS. The PSs are categorized by three basic structures (Fig. 2) such as porphyrin, chlorin, cyanine and other dyes (i.e., Methylene blue, toluidine blue, Rose Bengal and Hypericin). PSs also can be divided with different generation such as first, second and third generation. The next generation of PSs is being developed using carrier system (i.e., Liposome, nanoparticle and monoclonal antibody).

### First generation of PS

Hematoporphyrin and its derivatives are the first generation of PS. Hematoporphyrin was isolated from haemoglobin of red blood cells through concentrated sulfuric acid treatment in 1841 by Schere [24]. Later, hematoporphyrin was further purified in the form of photofrin [9]. First generation of PS has been widely used for treating different cancers in clinical. However, there was some limitation and intrinsic drawbacks such as poor chemical purity, short wavelength of light, prolonged half-life and intense accumulation in normal tissues, resulting photosensitive toxicity [25, 26].

### Photofrin®

**Fig. 3:**
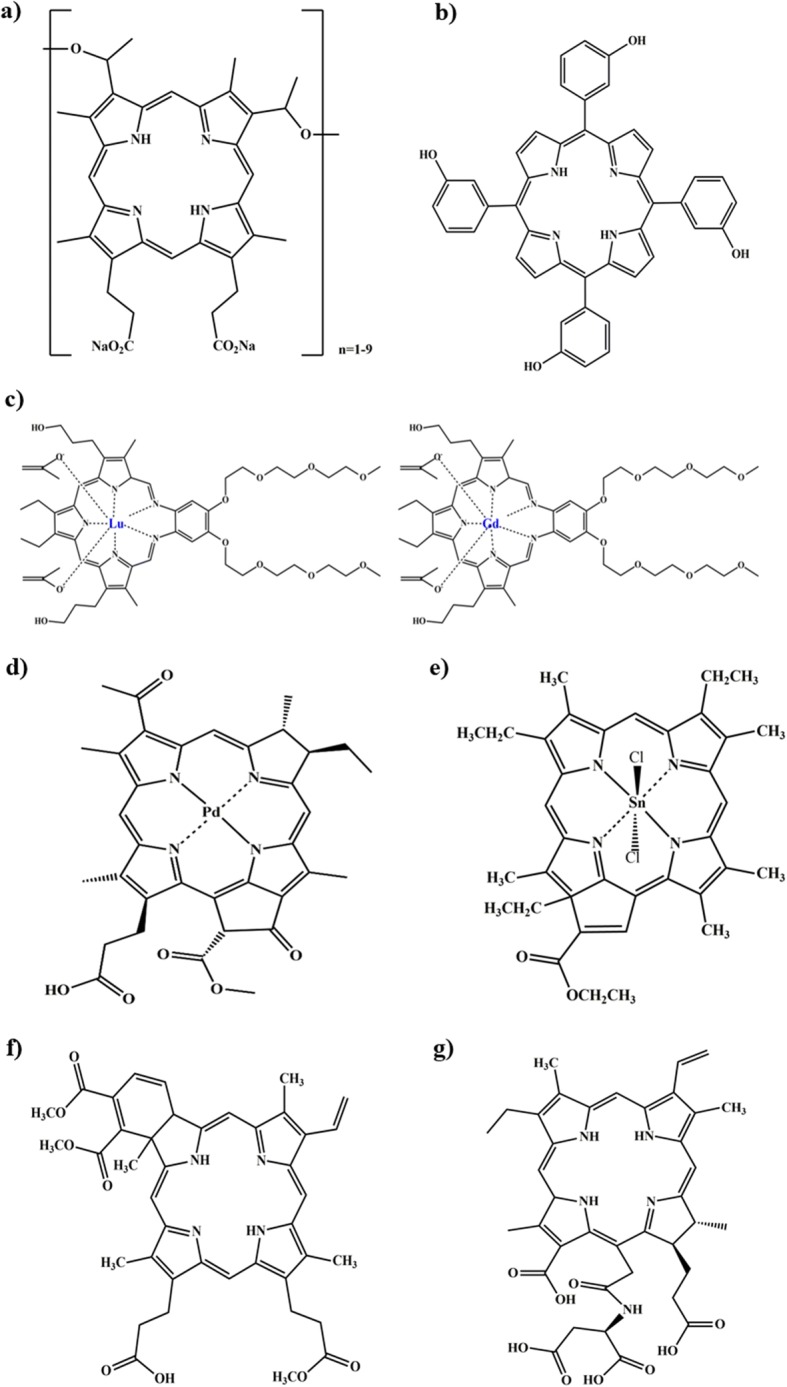
Chemical structure of clinically approved or under-clinical developing photosensitizing agents. **a**) Porfimer sodium (Photofrin®), **b**) Temoporfin (Foscan®), **c**) Motexafin lutetium and Motexafin gadolinium, **d**) Palladium bacteriopheophorbide, **e**) Purlytin®, **f**) Verteporfin (Visudyne®), **g**) Talaporfin (Laserphyrin®)

Photofrin® (Porfimer sodium; Axcan Pharma, Inc.) (Fig. 3a) was the first approved PDT agent for the treatment of obstructive esophageal cancer in 1995 [27]. It is still widely used for PDT for treatment of various cancers; lung cancer, bladder cancer, cervical cancer and etc. [28, 29]. Photofrin® is injected intravenously, readily accumulates in the tumour environment and irradiated with 630 nm wavelength laser light. Cellular damage caused by Photofrin® is a consequence of the propagation of photodynamic reaction. Photofrin® persists over 2 months after the administration [30]. During and after Photofrin® treatment, sunlight and other strong light exposure must be avoided. Photofrin® is commercially available in Canada, Japan, United States and European countries.

### Second generation of PS

Second-generation PSs have been improved in the purity, long wavelength absorption, photosensitivity and tissue selectivity. The second-generation PS are fulfilled with several serious drawbacks using first generation PS. First generation PS are not very specific to cancer cells and tend to accumulate in normal tissues as well. First generation PS are not clear rapidly from the human body and it has lack of sensitivity. The second-generation PS are effective and technically superior than first generation PS. Most of second generation PSs are based on porphyrin and chlorin structure. Core-modified second-generation PSs were designed for mitochondrial specific target. Second generation PSs are excited at a long wavelength; therefore, deeper light penetration improves the treatment efficacy. The second-generation PS have been developed over the decades, including Motexafin lutetium (Lutrin® and Lutex®; Pharmacyclics Inc), Temoporfin (Foscan®; Biolitec AG), Palladium bacteriopheophorbide (Tookad®; Negma-Lerads), purpurins (Purlytin®), Verteporfin (Visudyne®; Novartis) and protoporphyrin IX precursors (Hexvix®, Metvix® and Levulan®) (Table 1).

**Table 1:** Overview of clinically approved and under clinical trials PSs

	Generic name	Excitation Wavelength	Manufacturer	Application
First generation	Clinically approved			
	Photofrin®	630	Axcan Pharma, Canada	Esophageal cancer, Lung adenocarcinoma, Endobronchial cancer
Second generation	Ameluz®/Levulan®	635	DUSA, USA	Mild to moderate actinic keratosis
	Metvix®/Metvixia®	570–670	Galderma, UK	Non-hyperkeratotic actinic keratosis and basal cell carcinoma
	Foscan®	652	Biolitec, Germany	Advanced Head and neck cancer
	Laserphyrin®	664	Meiji Seika, Japan	Early centrally located lung cancer
	Visudyne®	690	Novartis, Switzerland	Age-related macular degeneration
	Redaporfin®	749	Luzitin, Portugal	Biliary tract cancer
	Under clinical trails			
	Fotolon	665	Apocare Pharma, Germany	Nasopharyngeal, sarcoma
	Radachlorin	662	Rada-pharma, Russia	skin cancer
	Photochlor	664	Rosewell Park	Head and neck cancer
	TOOKAD	762	Negma-Lerads	Prostate cancer
	Antrin	732	Pharmacyclics	coronary artery disease
	Photrex	664	Miravant, USA	AMD
	Talaporfin	664	Meiji Seika, Japan	colorectal neoplasms, Liver metastasis

### Foscan®

Foscan® (Temoporfin; Biolitec) (Fig. 3b) is a second-generation photosensitizing agent, extensively used for treatment of head and neck cancer [31, 32]. Foscan® was also selected for the treatment of breast and pancreatic cancer [33–35]. In 2002, Temoporfin was the first sensitizer used for prostate cancer after radiotherapy in a clinical study. Patients were monitored with prostate specific antigen (PSA) measurements and prostate biopsies. PDT study was conducted with fourteen patients using Foscan®. PSA decreased in nine patients and necrosis involved up to 91% of the prostate cross section [36, 37]. It presents higher tumour selectivity when light excited at specific wavelength of 652 nm. Light must be delivered not less than 90 h and not more than 110 h after Foscan® injection. Multiple course of treatment may be given at the discretion of physician to patients, however, Foscan® recommended minimum interval of 4 weeks between treatments. Clinical study was reported with 35 patients treated with Foscan® for head and neck cancer. PDT resulted in local control achieved up to 60% of patients. The recurrence-free survival rate was more than 50% for all patients almost 1 year [38]. The treatment of Foscan® may include eye and skin preservation for 6 weeks after the injection. Therapeutic effect of Foscan® is mediated through the generation of ROS in the presence of specific wavelength of light source. Most common side effects with Foscan are headache, haemorrhage, dysphagia and oedema.

### Lutex®

Lutex® (Motexafin lutetium, Pharmacyclics Inc) is a porphyrin-based PS used for the treatment of prostate cancer (Fig. 3c). Motexafin lutetium is activated with long wavelength light source with absorption range between 730 and 770 nm, due to macrocyclic modification. In 2008, a phase I clinical trial investigating the effect of Motexafin lutetium was performed against seventeen patients. This study used a wide variation of PS dose, light dose and PS-light interval. The patient treated with high dose of Motexafin lutetium (2 mg/kg), the PSA levels increased at initial and rapidly dropped to baseline. In contrast, low dose of Motexafin lutetium (0.5 mg/kg), did not fall below baseline of PSA level. Suggesting that high dose of Lutex® is promising than low dose for PDT [39]. Clinical trials observed the evidence of PDT-induced photobleaching in prostate cancer with pre and post treatment of Motexafin lutetium [40].

In another, Motexafin gadolinium (Xcytrin) is expanded metalloporphyrin PS for the treatment of brain metastasis and lung cancer [41, 42]. Motexafin gadolinium target to tumour cell than normal cell. It generates ROS through the intracellular oxygen and disrupts redox-dependent pathways triggers cell death through apoptosis. Sixty patients were treated with Motexafin gadolinium daily (4.4 mg/kg) for 5 consecutive days per week for intrinsic pontine gliomas. Patients were received intravenous bolus of Motexafin gadolinium and irradiated with standard dose. Clinical trial resulted in 18% of one-year event-free survival and 53% of overall survival. The addition of Motexafin gadolinium did not improve the survival of pediatric patients for 6 weeks with standard irradiation [43, 44]. But it was not approved from FDA for non-small cell lung cancer patients with brain metastases [41].

### Tookad®

Tookad® (Palladium bacteriopheophorbide, Negma Lerads/Steba Biotech) (Fig. 3d) is a second-generation PS and it is commonly used as vascular targeted PDT [45–47]. Tookad® is activated at a relatively long wavelength of 762 nm which permits deep tissue penetration. After light activation, Tookad® undergoes systemic circulation and leads to the intravascular generation of super oxide and hydroxyl radicals and kills cancer. Several studies reported safety evaluation with no serious adverse effect [48, 49]. Tookad® was tried for the treatment prostate cancer in phase II and III [50, 51]. For the treatment of prostate cancer, Tookad® is administered as a single dose intravenous injection for 10 min. Tookad® has the practical advantage that light may be instantaneously delivered during or just after injection. Tookad® is accumulated selectively in tumour blood vessels and quickly cleared from the body within short time. Tookad has fast clearance (half-life ~ 0.02–0.03 h) and it is retained in the tumour vascular until clearance and then induce tissue phototoxic, leading to tumour vessel destruction and death [52]. Higher dose of Tookad® can cause skin sensitivity, but it is greatly reduced by quick elimination rate [53].

### Purlytin®

Purlytin® is a chlorin based PS. Phase I/II clinical trials was performed for the treatment of breast cancer and Kaposi’s sarcoma in patients. Three patients were treated with a single dose Purlytin® (Tin ethyl etiopurpurin, Fig. 3e) for Kaposi’s sarcoma. One day later, the patients were exposed to a laser at 664 nm. Effect of Purlytin® was maintained up to 6 months [54]. In 1998, a phase II/III clinical study was performed for treatment of breast cancer. After 6 months follow-up, complete response was achieved over 90% patients. There was no observed systemic toxicity [55]. Putrlytin® has drawback of dark toxicity and photosensitivity.

### Visudyne®

Visudyne® (Verteporfin, Novartis) is a benzoporphyrin derivative of verteporfin (Fig. 3f) Verteporfin therapy is considered as a first-line therapy for serious ocular diseases; age-related macular degeneration and myopic choroidal neovascularization [56, 57]. For the cancer treatment, Verteporfin is administrated by intravenously and activated by red-shifted and intensified laser absorption with 690 nm wavelength. The elimination half-life of Verteporfin is approximately 5–6 h. The tissue penetration is 50% greater than photofrin® under the activation of long wavelength (690 nm). After verteporfin PDT frequently reported injection site reactions, including pain, oedema, inflammation, haemorrhage, discolouration. Patients were resulted visual impairment such as blurred, fuzzy vision, photopsia, reduced visual acuity and visual field defects, including scotoma and black spots. Verteporfin efficiently induced tumour necrosis even in the advanced pancreatic cancer [58].

### Laserphyrin®

Laserphyrin® (Talaporfin) is a mono-L-aspartyl chlorin and it was approved in Japan in 2004 as a PDT for lung cancer. Laserphyrin® was also employed for early head and neck cancer patients. Laserphyrin® (Fig. 3g) is injected intravenously, irradiated superficially with laser at wavelength of 664 nm. Tumour tissue samples were obtained for the measurement of fluorescence intensity and Magnetic Resonance Imaging (MRI). They marked the boundary between tumour (contrast) and normal tissue (non-contrast). Contrast enhanced region exhibited strong fluorescence intensity than non-contrast enhanced regions, confirmed an increasing trend of fluorescence intensity within tumour cell [59, 60]. Talaporfin PDT achieved better therapeutic response rate about 80% in over the year. Talaporfin is going through the phase II trial for the treatment of colorectal neoplasms and liver metastasis.

### Third generation

PDT has uncovered a wide variety of agents some of which are effective at high level oxygen release and targeting cancer cells, with less targeting of healthy ones. Third generation photosensitizer is being developed, but still not fertile. The second-generation PSs has several critical issues such as poor water-solubility, body clearance rate and photo-bleaching. Second-generation PSs did not show enough tumour selectivity. Many studies focus on third generation PSs that shows higher tumour specificity with long-wavelength light activation. This can be achieved by conjugation or encapsulation of exist PS in carriers that can delivered to the target tissue [61–63]. Novel third-generation PS conjugation with antibodies are developed for specific tumour tissue target [64, 65].

Development of PS are still do not have the fully finished literature or research and it seems early days on deeper located cancers. Downsides of the earlier PS drugs were that they do have side-effects such as allergic reactions, nausea and inflammation etc. [66]. They also can interlock on to surrounding healthy cells and kills simultaneously. Most exciting PS are developing from the natural agents such as chlorophyll compounds allows penetration into deeper levels in the body, which has a similar structure of haemoglobin [67]. Thus, it can circulate in the blood stream to almost any location. They don’t need deeper penetration of light; cancer cells are simultaneously targeted throughout the body. It may be able to knock out cancer cells all over the body with target delivery and not just the primary tumour but all the secondaries type of cancer.

## Conclusions

Despite of many advanced research and preclinical studies the translational status of PDT remains unsatisfactory. In any cases, the key source of PDT should be optimized by adjustment of parameters such as input dose, intra-tumoural drug levels, light source, and tissue oxygen condition. The ideal PS are chemically pure, miscible and stable in body fluids. And it is necessary to develop novel PSs with multifunctional capability for advanced cancer therapy. We have attempted to provide an information of currently developed and clinically available PSs. Development of a versatile with efficient PS composes are applicable for bioimaging and PDT in the future. As reviewing the various PDT techniques and potential strategies for enhanced photodynamic effects, it is expected that this information can offer the direction for the development of next generation of PDT reagent.
